# Antibody Responses and the Effects of Clinical Drugs in COVID-19 Patients

**DOI:** 10.3389/fimmu.2021.580989

**Published:** 2021-06-09

**Authors:** Liwen Wei, Yuqi Shang, Xi Liu, Xinghua Li, Gongqi Chen, Siping Liang, Zhengyu Zou, Tao Ding, Zhongsi Hong, Minhao Wu, Jinyu Xia

**Affiliations:** ^1^ Program of Infectious Diseases, The Fifth Affiliated Hospital of Sun Yat-sen University, Zhongshan School of Medicine, Sun Yat-sen University, Guangzhou, China; ^2^ Department of Infectious Diseases, The Fifth Affiliated Hospital of Sun Yat-sen University, Zhuhai, China; ^3^ Key Laboratory of Tropical Disease Control, Ministry of Education, Sun Yat-sen University, Guangzhou, China; ^4^ Department of Immunology, Zhongshan School of Medicine, Sun Yat-sen University, Guangzhou, China; ^5^ Biosafety Laboratory, Zhongshan School of Medicine, Sun Yat-sen University, Guangzhou, China

**Keywords:** COVID-19, SARS-CoV-2, antibody, chloroquine, humoral response

## Abstract

The coronavirus disease 2019 (COVID-19) emerged around December 2019 and have become a global epidemic disease currently. Specific antibodies against SAS-COV-2 could be detected in COVID-19 patients’ serum or plasma, but the clinical values of these antibodies as well as the effects of clinical drugs on humoral responses have not been fully demonstrated. In this study, 112 plasma samples were collected from 36 patients diagnosed with laboratory-confirmed COVID-19 in the Fifth Affiliated Hospital of Sun Yat-sen University. The IgG and IgM antibodies against receptor binding domain (RBD) and spike protein subunit 1 (S1) of SAS-COV-2 were detected by ELISA. We found that COVID-19 patients generated specific antibodies against SARS-CoV-2 after infection, and the levels of anti-RBD IgG within 2 to 3 weeks from onset were negatively associated with the time of positive-to-negative conversion of SARS-CoV-2 nucleic acid. Patients with severe symptoms had higher levels of anti-RBD IgG in 2 to 3 weeks from onset. The use of chloroquine did not significantly influence the patients’ antibody titer but reduced C-reaction protein (CRP) level. Using anti-viral drugs (lopinavir/ritonavir or arbidol) reduced antibody titer and peripheral lymphocyte count. While glucocorticoid therapy developed lower levels of peripheral lymphocyte count and higher levels of CRP, lactate dehydrogenase (LDH), α-Hydroxybutyrate dehydrogenase(α-HBDH), total bilirubin (TBIL), direct bilirubin (DBIL). From these results, we suggested that the anti-RBD IgG may provide an early protection of host humoral responses against SAS-COV-2 infection within 2 to 3 weeks from onset, and clinical treatment with different drugs displayed distinct roles in humoral and inflammatory responses.

## Introduction

The coronavirus disease 2019 (COVID-19) emerged around December 2019 (1). From now on, there have been over one billion confirmed cases of COVID-19 (2). Although the epidemic situation in China has been reduced, the international situation is still not optimistic. The infectious pathogen has been identified a SARS-related coronavirus and named as SARS-COV-2.

Viral-specific antibodies play an important role in blocking viral infection. The SAS-COV-2 specific antibodies could be detected in patients’ serum or plasma, and has been used as a diagnose marker for COVID-19 (3, 4). Moreover, transfusion plasma or serum from recovered patients has been applied as a potential therapy in clinical trials (5). Fan Wu et al. detected specific antibodies against SARS-COV-2 in COVID-19 patients within three weeks from onset, and found that elderly and middle-age patients had significantly higher plasma neutralizing Ab titers (6). However, whether the antibody titers correlated with disease progression has not been fully explored.

Currently, chloroquine, antiviral drugs like lopinavir/ritonavir, as well as glucocorticoids have been widely used in COVID-19 therapy. Several studies demonstrated that chloroquine may be a cost-effective therapy for combating the COVID-19 pandemic (7, 8). Current reports indicate that SARS-COV-2 infection may affect the heart muscle and cause myocarditis (9). Therefore, treatment of COVID-19 patients with chloroquine requires more cautions, because of its cardiotoxicity. The role of lopinavir/ritonavir, arbidol and glucocorticoids therapy on COVID-19 has also been challenged, for their limited effectiveness, as well as side effects like vomiting, diarrhea, or bone necrosis in some cases (10, 11). So far, little is known regarding the role of these clinical drugs on specific humoral responses in COVID-19 patients.

In the present study, ELISA assay was applied to measure SARS-Cov-2 specific IgG/IgM antibodies in plasma from COVID-19 patients, and the correlation between antibody titer and the time of positive-to-negative conversion or disease severity were analyzed. Moreover, clinical indicators from patients grouped by medication were collected and analyzed. The results may provide useful information for clinical medication to treat COVID-19 pandemic.

## Methods

### Ethics Approval

This study was approved by the Institutional Review Board of the Fifth Affiliated Hospital of Sun Yat-sen University (Zhuhai, China) [No. ZDWY (2020) Lunzi No. (K22-1)] and the Guangdong Natural Science Fund for Distinguished Young Scholars (2016A030306004). All participants have signed a written informed consent.

### Patients

The study included a cohort of 36 COVID-19 patients admitted to the Fifth Affiliated Hospital of Sun Yat-sen University. We diagnosed COVID-19 in accordance with the criteria in the WHO interim guidelines and the Diagnosis and Treatment Plan for Novel Coronavirus Pneumonia by the National Health Commission (seventh trial version) (12). A confirmed case was defined as a positive result of SARS-CoV-2 for throat swab specimens by real-time PCR. Clinical information of enrolled patients, including complete blood counts, blood biochemistry, and chest imaging results were obtained from clinical record. None of the patients diagnosed as severe disease were admitted to the ICU. The treatment of chloroquine, Lopinavir/Ritonavir, or glucocorticoid was according to the medical advice. The detailed information and clinical characteristics of 36 COVID-19 patients were shown in **Tables 1** and **2**.

**Table 1:** Clinical information of COVID-19 patients.

Sample ID	Type	Days from symptom onset to antibody test	Treatment	Days of nucleic acid positive-to-negative conversion	Days of re-detectable positive nucleic acid from symptom onset
P1	Severe	10,17,55	C+A+G	10	N/A
P2	Severe	11,18,34	C+A+G	13	N/A
P3	Severe	9,24,35,44	C+A+G	15	N/A
P4	Severe	18,27,33	C+A	12	N/A
P5	Severe	18,24,36,54,61	C	29	30
P6	Mild	14,20,29,42,49	C+A	29	N/A
P7	Mild	14,20,28,41	C+A	22	21
P8	Mild	6,13,18	A	6	N/A
P9	Mild	13,27,41,49,56	C+A	15	N/A
P10	Mild	9,16,45,52	C+A	16	N/A
P11	Mild	5,14,38,44	C+A	16	N/A
P12	Mild	11,20,38	A	8	N/A
P13	Mild	2,9,16,44,55	C+A	11	N/A
P14	Mild	8,15,22,31,41	C+A+G	22	17
P15	Mild	11,17,47,54	C+A	32	N/A
P16	Severe	17,21,52	C+A+G	8	N/A
P17	Severe	8,16	C+A	9	N/A
P18	Severe	13,19	C+A	11	N/A
P19	Severe	11,17	C+A	10	N/A
P20	Severe	15,22,27	C+A	11	N/A
P21	Severe	5,41	N/A	4	N/A
P22	Mild	15,41,63	A	15	14
P23	Mild	30,41,47	C+A	27	25
P24	Mild	40,48	C	6	N/A
P25	Mild	51,58	C+A	20	37
P26	Mild	7,21,29	C+A	1	N/A
P27	Mild	14,28	C+A	1	N/A
P28	Mild	7,28	C	5	N/A
P29	Mild	6,19,27	C	2	N/A
P30	Mild	25,43,49	C	33	26
P31	Mild	17,21,50	C+A	13	N/A
P32	Mild	45,51	A	11	N/A
P33	Mild	5,10,20	C	N/A	N/A
P34	Mild	7,14	C	1	N/A
P35	Mild	16,48	A	12	N/A
P36	Mild	12,35,42	C+A	8	N/A

C, Chloroquine; A, Anti-viral drugs(Lopinavir/ritonavir or Arbidol); G, Glucocorticoid; P, patient; N/A, not applicable. Days of nucleic acid positive-to-negative conversion is calculated from the time of admission.

**Table 2:** Clinical characteristics of COVID-19 patients.

	Severe disease (n=11)	Mild disease (n=25)	p Value
**Age, Years**	64(36-80)	49(16-71)	
**Sex**			
male	4(36%)	12(48%)	0.72
female	7(64%)	13(52%)	
**Comorbidities**			
Cardiovascular disease	5(45%)	4(16%)	0.10
Respiratory diseases	1(9%)	2(8%)	>0.99
Digestive system disease	0(0%)	3(12%)	0.54
Endocrine system disease	4(36%)	3(12%)	0.17
Tumor	0(0%)	1(4%)	>0.99
hypertension	5(45%)	3(12%)	0.04
diabetes	3(27%)	2(8%)	0.15
none	4(36%)	14(56%)	0.47
**Presenting symptoms**			
Fever	10(91%)	15(60%)	0.12
cough	4(36%)	13(52%)	0.48
Dyspnoea	2(18%)	0(0%)	0.09
Sore throat	1(9%)	4(16%)	>0.99
Chest discomfort	0(0%)	1(4%)	>0.99
Headache	1(9%)	4(16%)	>0.99
Myalgia	2(18%)	3(12%)	0.63
Malasie	2(18%)	1(4%)	0.22
Nausea	0(0%)	0(0%)	>0.99
Diarrhoea	1(9%)	2(8%)	>0.99
none	0(0%)	4(16%)	0.29
**Nucleic acid positive in feces**	6(55%)	16(64%)	0.72
**Time from onset to admission (days)**	5±1.34	4.±0.94	0.50
**Hospitalization time (days)**	20±2.06	24±1.70	0.16
**Time from onset to discharge (days)**	24±2.93	27±2.05	0.41
**Chest CT**			
viral pneumonia	10(91%)	18(72%)	0.39
chronic inflammation	0(0%)	2(8%)	>0.99
bronchitis	0(0%)	1(4%)	>0.99
others	1(9%)	1(4%)	0.52
none	0(0%)	3(12%)	0.54
Number of infected lobes (0-1)	1(9%)	10(40%)	0.12
Number of infected lobes (2-5)	10(91%)	15(60%)	0.12
**Blood Test**			
White blood cell count(10^9/L)	4.94±0.97	5.39±0.66	0.69
Red blood cell count(10^12/L)	3.97±0.37	4.02±0.27	0.92
Hemoglobin(g/L)	124.00±9.86	112.60±7.61	0.37
Hematocrit(%)	35.68±2.90	34.14±2.70	0.23
Platelet count(10^9/L)	240.00±33.85	310.10±39.90	0.21
Neutrophil count(10^9/L)	2.92±0.74	3.18±0.50	0.77
Lymphocyte count(10^9/L)	1.38±0.23	1.52±0.21	0.66
Monocyte count(10^9/L)	0.50±0.07	0.57±0.11	0.64
Eosinophil count(10^9/L)	0.11±0.06	0.09±0.03	0.82
Alanine aminotransferase(U/L)	38.20±16.84	29.94±6.33	0.59
Aspartate aminotransferase(U/L)	27.10±6.81	23.27±3.14	0.57
Total bilirubin(μmol/L)	12.73±1.96	12.11±2.82	0.88
Direct bilirubin(μmol/L)	5.38±0.62	5.54±1.30	0.93
Total protein(g/L)	77.11±2.50	74.66±1.51	0.40
Albumin(g/L)	39.28±0.72	41.85±0.56	0.02
Lactate dehydrogenase(U/L)	162.50±10.97	162.90±10.82	0.98
alpha-hydroxybutyrate dehydrogenase(U/L)	127.80±6.58	131.60±9.00	0.75
Creatine kinase(U/L)	36.25±5.79	75.80±22.78	0.18
Creatine Kinase Isozyme MB(U/L)	9.60±1.49	14.18±3.11	0.26
C-reactive protein(mg/L)	5.86±1.19	2.93±1.03	0.10
Blood urea nitrogen(mmol/L)	4.00±0.58	4.52±0.40	0.47
Blood creatinine(μmol/L)	67.85±4.76	68.38±7.49	0.96
The corrected oxygen partial pressure (mmHg)	98.65±28.41	92.06±4.39	0.80
CO2 partial pressure after correction(mmHg)	43.80±2.05	40.32±1.32	0.18
Corrected pH	7.39±0.02	5.95±1.46	0.41
Whole blood lactic acid(mmol/L)	1.58±0.13	1.54±0.19	0.89

### ELISA

Recombinant protein of SARS-CoV-2 receptor binding domain (RBD) or S1 protein (Sino biological, Beijing, China) was coated at 2 μg/ml on a 96-well ELISA plate overnight at 4°C. Wells were blocked with 5% non-fat milk in PBST (PBS with 0.05% Tween-20) at 37°C for 30 minutes. Next, the plate was wash with PBST for 3 times. Then, the plate was incubated with 1:100 diluted plasma in PBS at 37°C for 30 minutes. A 1:2500 dilution of horseradish peroxidase (HRP)-conjugated goat anti-human IgG antibody (Invitrogen, Carlsbad, CA) or a 1:5000 dilution of Goat-anti-Human IgM HRP (Sigma, St. Louis, MO) was respectively added to the plate and incubate at 37°C for 30 minutes. Wells were wash with PBST for 3 times between each step. At last, TMB (Beyotime, Shanghai, China) was added to the wells and react for 10min. The plates were read at 450nm (Bio-Tek, ELx808). The ELISA test in our study were repeated twice and phosphate buffer saline was used as the negative control of plasma.

### Statistical Analysis

Statistical analyses were carried out using GraphPad Prism 7.0. We compared categorical variables using Fisher’s exact test. Differences between two groups were analyzed using paired or unpaired t test, while differences between ≥3 groups were compared by one-way analysis of variance (ANOVA). Correlations were calculated using standard Pearson correlation. A p value less than 0·05 was judged as statistically significant. Data were considered statistically significant at p<0.05.

## Results

### Patient Characteristics

The present study included a cohort of 36 COVID-19 patients admitted to the Fifth Affiliated Hospital of Sun Yat-sen University between Jan 22, 2020 to March 7, 2020. Until May 28th, all patients have been cured and discharged. Among 36 patients, 11 patients had severe symptoms and 25 patients had mild symptoms (including 4 patients who did not have any symptoms), and the median age of patients was 56 (range 21-80). Seventeen patients had underlying diseases and the most common diseases were Cardiovascular disease (in 9 patients, 25%) and hypertension (in 8 patients, 22%). The most common symptom was fever in 25 patients (69%). Viral nucleic acid can be detected in throat swab specimens of all the 36 patients (100%), and in the feces of 21 patients (60%). The patient’s lung imaging report is one of the important indicators to judge virus infection. Viral pneumonia, chronic inflammation or bronchitis were diagnosed in 33 patients (91%). Lung consolidation is an important imaging indicator of COVID-19 and the number of consolidation lung lobes indicates the disease severity. The number of consolidation lung lobes are limited to 1 (0-1 lobe), or more than 1 (2-5 lobes). In our study, 91% of patients with severe symptoms and 60% of patients with mild symptoms had consolidated areas in the lungs more than 1 lobe. None of the patients diagnosed as severe disease were admitted to the ICU, so their clinical indicators were almost common. Based on the blood test results, the level of albumin was higher in patients with mild vs severe symptoms (P=0.02).

### COVID-19 Patients Developed SARS-CoV-2 Specific Antibodies

**Figure 1:**
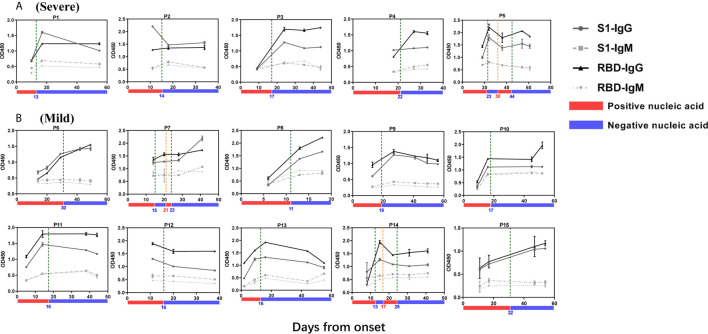
SARS-CoV-2-specific Antibodies Increases During Disease Progression. Plasma were collected at different time points after syndrome onset. The titers of IgG or IgM antibody binding to S1 protein or RBD in five COVID-19 patient plasma with severe symptom **(A)** or ten patients with mild symptoms **(B)** are shown. The green dotted line represents the time point when the patient’s nucleic acid turns negative while the orange dotted line represents the time point when the patient’s nucleic acid turns positive. The red process bar indicates that the patient’s nucleic acid is positive, and the blue indicate a negative result.

In total, 110 plasma samples were collected from 36 patients during their hospitalization period. The S protein has two parts, subunit 1 (S1) and subunit 2 (S2). The receptor binding domain (RBD), which mediates the virus and host cell surface interaction, is located on S1. Therefore, we measured the antibodies, including IgG and IgM, against S1 protein or its’ RBD in the plasma samples using ELISA. The IgG level against S1 protein and RBD increased in COVID-19 patients compared with healthy people (**Supplementary Figure 1**). After the patients’ nucleic acid in throat swab turned negative, they entered the clinical observation period. However, during this period, SARS-COV-2 re-infected several recovered patients. The nucleic acid in their throat swab become positive again. In **Figure 1**, the green and orange dashed line points the time point of viral nucleic acid turning negative and positive, respectively. We also used bars in different colors to indicate the results of patient’s nucleic acid test. The red bar indicates that the patient’s nucleic acid is positive, and the blue indicate a negative result. As shown in **Figure 1**, the antibody titer of anti-S and anti-RBD IgM and IgG increased during the first three weeks from disease onset in most patients.

### The Time of Virus Nucleic Acid to Negative Was Negatively Correlated With Anti-RBD IgG Levels

**Figure 2:**
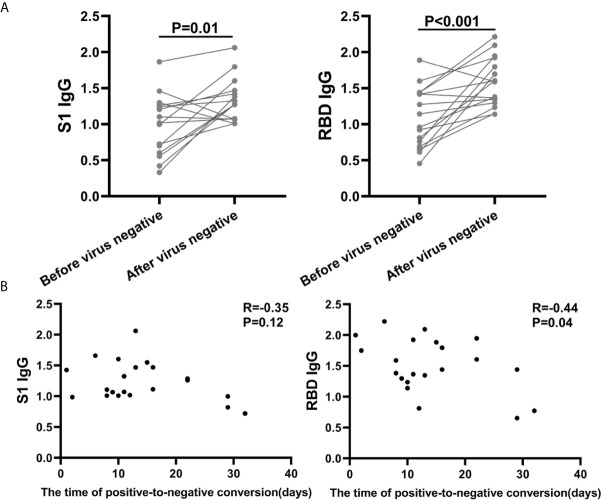
The Time of Positive-to-negative Conversion of SARS-CoV-2 Nucleic Acid was Negatively Correlated with Antibody Titer. **(A)** Comparison of the titers of IgG antibodies before and after the throat swab nucleic acid turn to negative. The antibody used for analysis was obtained within 9 days before and after the virus turned negative (n = 16). **(B)** Correlation analysis of time of positive-to-negative conversion and IgG antibody titers (n = 21). The titers of IgG antibody against S1 protein or RBD between 8-21 days from symptom onset were collected and analyzed.

In the present study, we regard the day when the patients’ nucleic acid in throat swab turned negative as a time point. We analyzed the difference in antibody titers of 16 patients within nine days before and after this time point. We found that the S1 and RBD IgG antibody titer increased significantly after the patients’ nucleic acid turns negative (**Figure 2A**). In clinic, RT-PCR was used to detect nucleic acid Ct value which indicate the virus load. We collected nucleic acid Ct value in throat swab of each patient during their hospitalization period. The nucleic acid Ct value in throat swab was positively correlated with the anti-S1 and anti-RBD IgG antibody titer (**Supplementary Figure 3**). However, some of the Ct value were beyond detection. Therefore, we analyzed the time of positive-to-negative conversion of SARS-COV-2 nucleic acid in throat swab specimens and the IgG antibody titer and found that they have a negative correlation (**Figure 2B**). In summary, these data suggested that the anti-S1 and anti-RBD IgG antibody enhanced the host humoral defense against SARS-COV-2 infection.

### Patients With Mild Symptoms Had Higher Anti-RBD IgG Levels After Two Weeks From Onset

**Figure 3:**
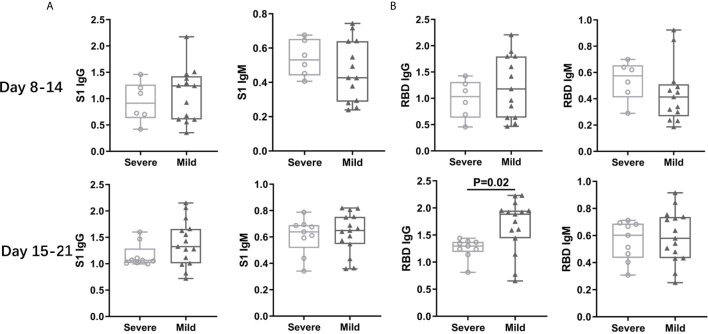
Patients with Mild Symptoms Had Higher Level IgG after Two Weeks from Onset. The patients were grouped by disease symptom. The titers of IgG and IgM antibody against S1 protein **(A)** or RBD **(B)** between 8-21 days from symptom onset were collected and analyzed.

Next, we analyzed the differences of antibody titers between COVID-19 patients with severe vs mild symptoms. We compared the antibody titers in different periods from onset and found that anti-RBD IgG titers in the mild group were higher than those in the severe group after two weeks from onset (**Figure 3**), whereas the antibody titers were comparable in the two groups in other periods (**Supplementary Figure 4**). These results indicated that high level of specific antibodies might be useful to clear the viruses and to alleviate the patients’ symptoms.

**Figure 4:**
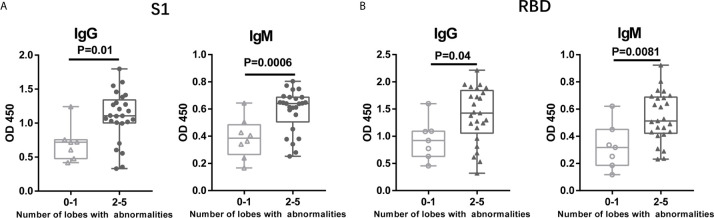
Patients with More Lung Lobes with Abnormalities Developed Higher Levels of Antibodies. The patients were grouped based on the number of based on the number of lung lobes with abnormalities, limited to 1 lobe (0-1), or involved more than 1 lobe (2-5). The plasma samples were collected on the day when the patients detect chest CT. The antibody titers of IgG and IgM antibody against S1 **(A)** protein or RBD **(B)** were collect and analyzed.

After infected by SARS-COV-2, chest computed tomography (CT) imaging is an essential component of evaluations. Chest CTs generally showed small patchy shadows and interstitial lung disease, which further developed into ground glass attenuation and infiltration. Pulmonary consolidation occurred in severely affected patients; however, pleural effusion was relatively rare. We grouped the patients based on the number of lung lobes with abnormalities, limited to 1 lobe (0-1), or involved more than 1 lobe (2-5). We found that when the patients had more lung lobes with abnormalities, they tend to have a higher antibody titer against SARS-COV-2 (**Figure 4**).

### The Effects of Clinical Drugs in COVID-19 Patients’ Humoral Immune Response and Clinical Indicators

**Figure 5:**
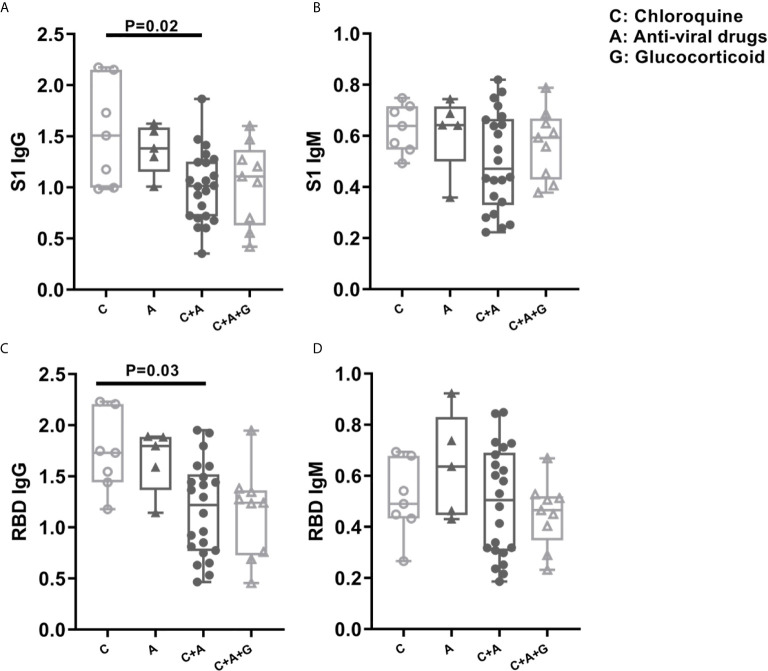
The Difference of Patients’ Antibody Titer after Chloroquine, Anti-viral Drug or Glucocorticoid Medication. The patients were grouped in chloroquine group, antiviral drug group, chloroquine plus antiviral drugs, chloroquine, antiviral drugs plus glucocorticoids group. The titers of IgG and IgM antibody against S protein **(A, B)** or RBD **(C, D)** between 8-21 days from symptom onset were collected and analyzed. C, Chloroquine; A, Anti-viral Drug; G, Glucocorticoid.

**Figure 6:**
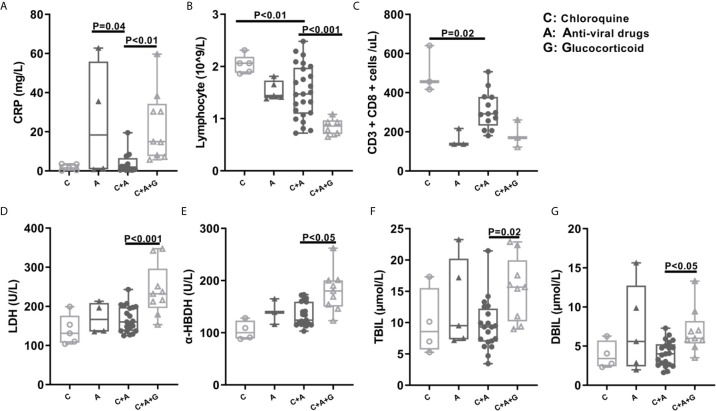
The Difference of Patients’ Clinical Indicators after Chloroquine, Anti-viral Drug or Glucocorticoid Medication. The patients were grouped in chloroquine group, antiviral drug group, chloroquine plus antiviral drugs, chloroquine, antiviral drugs plus glucocorticoids group. Several clinical indicators like CRP **(A)**, lymphocyte **(B)**, CD3+CD8+ cells **(C)**, LDH **(D)**, α-HBDH **(E)**, TBIL **(F)**, DBIL **(G)** collected between 8-21 days from symptom onset were analyzed. α-HBDH, α-Hydroxybutyrate dehydrogenase; CRP, C-reaction protein; LDH, Lactate dehydrogenase; TBIL, Total bilirubin; DBIL, Direct bilirubin; C, Chloroquine; A, Anti-viral Drug; G, Glucocorticoid.

Currently, some virus-targeted drugs, small molecule inhibitors or corticosteroids have been used to treat COVID-19 patients. In the present study, some patients received clinical treatments with combined drugs (e.g. Chloroquine, antiviral drug and glucocorticoids). Therefore, we divided the patients into four group as following: 1) chloroquine group used only chloroquine (abbreviation: C); 2) antiviral drug group used only lopinavir/ritonavir and arbidol (abbreviation: A); 3) chloroquine plus antiviral drugs were those used chloroquine, lopinavir/ritonavir and arbidol (abbreviation: C+A); 4) chloroquine, antiviral drugs plus glucocorticoids were those used all three medicines (abbreviation: C+A+G). While comparing group A with group C+A, we found that the use of chloroquine did not significantly influence the patients’ antibody titer but reduced C-reaction protein (CRP) level (**Figures 5A–D**, **6A**). We also found that using anti-viral drugs (lopinavir/ritonavir or arbidol) reduced antibody titer and lymphocytes counts in COVID-19 patients by comparing group C and C+A (**Figures 5A, B**, **6B, C**). Moreover, the comparison of group C+A and group C+A+G demonstrated that glucocorticoid therapy caused lower levels of lymphocytes counts and higher levels of CRP, lactate dehydrogenase (LDH), α-Hydroxybutyrate dehydrogenase(α-HBDH), total bilirubin (TBIL), direct bilirubin (DBIL) (**Figures 6B**, **D–G**).

## Discussion

The patients will produce specific antibodies after being infected by the virus, so in addition to nucleic acid amplification, the detection of specific antibodies against SARS-CoV-2 is also an effective way to diagnose current or cured COVID-19 patients (3, 13). SARS-CoV-2 has four main structural proteins: spike protein (S), nucleocapsid (N), membrane protein (M), envelope protein (E). The S protein is a very important surface protein of the coronavirus, which is closely related to the infectious ability of the virus. N protein is abundant in coronaviruses and is a highly immunogenic protein that participates in genome replication and cell signaling pathway regulation. To KK et al. confirmed that more patients had earlier seropositivity for anti-RBD than anti-NP for both IgG and IgM, which means that the detection efficiency of anti-RBD antibodies is higher. Moreover, they also found that the rate of seropositivity for anti-RBD IgG is 100% in the patients with serum specimens available for 14 days or longer after symptom onset (13). Therefore, spike protein and its RBD are used in the present study and we found that all COVID-19 patients developed SARS-CoV-2-specific antibodies, including IgG and IgM against RBD and S1 protein.

Antibody responses in COVID-19 patients have been wildly studied in the past few months (14–16). In our study, we found the IgG antibody titers kept increasing during the course of disease progression in most cases, and reached the peak at 10 to 15 days after disease onset. The time for positive-to-negative conversion of SARS-CoV-2 nucleic acid was negatively correlated with the antibody titer of anti-RBD and anti-S1 IgG, indicating that the IgG antibodies enhanced host humoral defense against SARS-COV-2 and contributed to the clearance of viruses. These findings are consistent with previous reports. However, there is no obvious correlation between the IgM antibody titers and the SARS-COV-2 disease process or the viral load. We also found that patients with mild symptoms had higher anti-RBD IgG levels after one week from onset and the significance was appeared after 15-21 days from onset. Quan-Xin Long et al. detect specific antibodies against SARS-COV-2 and found that IgG and IgM titers in the severe group were higher than those in the non-severe group after 8-14 days from onset (15). In their study, patients in the severe group were all admitted in the ICU. We believe the susceptibility of these patients made them responded quickly after virus infection. As the disease progresses, for those patients who have non-critical diseases, higher antibody titer might be useful to alleviate the patients’ symptoms and beneficial to their recovery.

As the patients involved in the present study were not admitted to the ICU, most of patients had moderate symptoms. All of the severe patients did not have systemic respiratory failure. These patients had a relatively stable disease course, and therefore are suitable for medication research to determine the effects of different drugs on the patient’s humoral immunity. At present, there is no special medicine for treating COVID-19. Chloroquine is an immunomodulator used to treat malaria and some autoimmune diseases. While combating the COVID-19 pandemic, chloroquine can also be a cost-effective therapy (7, 8). A total of 197 patients completed chloroquine treatment, and 176 patients as historical controls with confirmed SARS-CoV-2 infection were enrolled in a multicenter prospective observational study (8). The scientists detected shorter median time to achieve an undetectable viral RNA in chloroquine group than in non-chloroquine. Hydroxychloroquine, a chemically modified chloroquine derivative, is considered to be less toxic. It is currently prescribed in combination with azithromycin for COVID-19 pneumonia. However, substantive clinical evidence questioned the side effects of this medication. The researchers argued that short-term hydroxychloroquine treatment may be safe, but addition of azithromycin could induce heart failure and cardiovascular mortality (9). More than one hundred thousand patients from five countries were included in this study. Moreover, a clinical multi-center trial from China also revealed that hydroxychloroquine is not effective for patients with mild disease recently. Patients treated with chloroquine often experienced side effects such as indigestion and vomiting (16). Researchers demonstrated that chloroquine may inhibited SARS-COV-2 infection in Vero cells because it could bind to the ganglioside domain of SARS-CoV-2 S protein (17). Others thought the iron starvation conditions induced by chloroquine may attenuate virus replication and induced the innate and adaptive immune responses (18). In our study, the results showed that the use of chloroquine did not significantly increase the antibody titers in COVID-19 patients, but reduced the levels of inflammatory injury markers such as CRP. Therefore, it demonstrated that the clinical effects of chloroquine in COVID-19 treatment may be due to its inhibitory role on inflammatory injury, rather than influencing the host humoral defense.

Clinically, anti-viral drugs, such as lopinavir/ritonavir and arbidol, have also been used to treat COVID-19 patients. Lopinavir/ritonavir is a protease inhibitor, which may inhibit the 3C like protease of SARS-CoV-2 and arbidol impedes trimerization of SARS-CoV-2 spike glycoprotein and inhibits host cell adhesion (19). It is reported that lopinavir/ritonavir or arbidol monotherapy may not improve the clinical efficacy of COVID-19 patients with mild to moderate symptoms (10, 20). Our data shown that the use of antiviral drugs was not conducive to the accumulation of antibodies and also reduced lymphocyte counts. We supposed that this maybe the one of the reasons why these drugs did not have good effects in clinical treatment for COVID-19. As therapies involving lopinavir/ritonavir and chloroquine or hydroxychloroquine had risks and uncertain benefits, currently they were excluded from COVID-19 treatment protocols. They should be used only in the context of clinical trials (21). However, researchers thought chloroquine or hydroxychloroquine could be prophylaxis of COVID-19 (22). In a world with vaccine shortage, such a precaution might be considered.

Glucocorticoids are mainly used to suppress excessive inflammation in Severe Acute Respiratory Syndrome (SARS), Middle East Respiratory Syndrome (MERS) or influenza A (H7N9) etc. (23, 24). During the epidemic of SARS in 2003, glucocorticoids were widely used in patients with severe symptom. A retrospective cohort study showed that glucocorticoids can reduce mortality and length of hospital stay of SARS (25). However, glucocorticoids also have a series of toxic side effects. Appling glucocorticoids in short-term, high-dose results in increased blood glucose, decreased lymphocytes or increased gastric acid secretion (11). Our results indicate that treatment with glucocorticoids significantly decreased the peripheral lymphocyte counts and enhanced the levels of damage indicators like LDH, α-HBDH in COVID-19 patients. This observation is consistent with the previous study showing that glucocorticoids treatment may affect lymphocytes and bring bone injury (26). Moreover, liver dysfunction markers like TBIL and DBIL were also enhanced in glucocorticoids-treated vs untreated patients, although their levels did not reach the level of clinical warning sign. In acute infection period, supplementing too much glucocorticoid will suppress immune defense, which is unfavorable for viral clearance. Therefore, we supposed that using glucocorticoid may not be suitable for mild patients and might affect patients’ liver function.

Glycosylation is one of the important post-translational modifications of proteins and the key quality attributes for evaluating antibodies. Glycosylation modification can improve antibody-dependent cell-mediated cytotoxicity (ADCC) and complement-dependent cytotoxicity (CDC). Therefore, studying the glycosylation of anti-SARS-COV-2 antibodies may help improve the clinical treatment.

In summary, we found that COVID-19 patients could produce specific IgG and IgM antibodies against SARS-COV-2 at a higher concentration 2-3 weeks after the symptom onset, and higher antibody titer might be useful to alleviate the patients’ symptoms and beneficial to their recovery. We analyzed the pattern of the antibody production and the effect of different drug treatment on antibody and patients’ clinical indicators. In the clinical treatment, choosing the appropriate dose and time point will help relieve the patient’s symptoms. Moreover, we should also pay attention to the patient’s humoral immune status and adjust the treatment plan. Our research provides a theoretical basis for the selection of drug treatment combination in clinic as well as the determination of drug treatment time.

## Data Availability Statement

The original contributions presented in the study are included in the article/**Supplementary Material**. Further inquiries can be directed to the corresponding authors.

## Ethics Statement

The studies involving human participants were reviewed and approved by Institutional Review Board of the Fifth Affiliated Hospital of Sun Yat-sen University (Zhuhai, China) (No. ZDWY [2020] Lunzi No. [K22-1]). The patients/participants provided their written informed consent to participate in this study.

## Author Contributions

LW, YS, XLiu contributed equally to this work. All authors contributed to the article and approved the submitted version.

## Funding

This work was supported by grants from National Natural Science Foundation of China [31970893], Guangdong Basic and Applied Basic Research Foundation [2020A1515010255], the Fundamental Research Funds for the Central Universities (19ykzd39; 19ykpy43), and the 111 Project [No. B12003, B13037], Open project of Key Laboratory of Tropical Disease Control (Sun Yat-sen University), Ministry of Education (2020kfkt08).

## Conflict of Interest

The authors declare that the research was conducted in the absence of any commercial or financial relationships that could be construed as a potential conflict of interest.

The reviewer YW declared a shared affiliation, with no collaboration, with the authors to the handling editor at the time of review.

## Supplementary Material

Supplementary Figure 1 COVID-19 Patients Developed SARS-CoV-2 Specific Antibodies. Comparison of The IgG and IgM antibody titer against S1 protein or RBD between COVID-19 patients (n=25) and healthy people (n=13). The antibody titers of COVID-19 patients were collected two weeks after symptom onset.

Supplementary Figure 2 Detection of IgG and IgM Antibody against S Protein or RBD in COVID-19 Patient. Plasma were collected at different time points after syndrome onset. The titers of IgG or IgM antibody binding to S1 protein or RBD in 21 COVID-19 patient plasma are shown. The green dotted line represents the time point when the patient’s nucleic acid turns negative while the orange dotted line represents the time point when the patient’s nucleic acid turns positive. The red process bar indicates that the patient’s nucleic acid is positive, and the blue indicate a negative result.

Supplementary Figure 3 The Nucleic Acid Ct Value in Throat Swab was Positively Correlated with the Anti-S and Anti-RBD IgG and IgM Antibody Titer. The titers of IgG or IgM antibody against S1 protein or RBD between 8-21 days from symptom onset and the nucleic acid Ct value in throat swab of each patient during their hospitalization period were collected. Fifty samples were included in this figure.

Supplementary Figure 4 The anti-S1 or anti-RBD IgG and IgM antibody titers were comparable in the severe and mild group between 8-63 days from symptom onset. The patients were grouped by disease symptom. The titers of IgG and IgM antibody against S1 **(A)** protein or RBD **(B)** between 8-63 days from symptom onset were collected and analyzed.
