# Supplementary figures and images for: Antibody Responses and the Effects of Clinical Drugs in COVID-19 Patients
Source: Front Immunol. 2021 Jun 9;12:580989. doi: 10.3389/fimmu.2021.580989 (PMC8220093; doi:10.3389/fimmu.2021.580989)

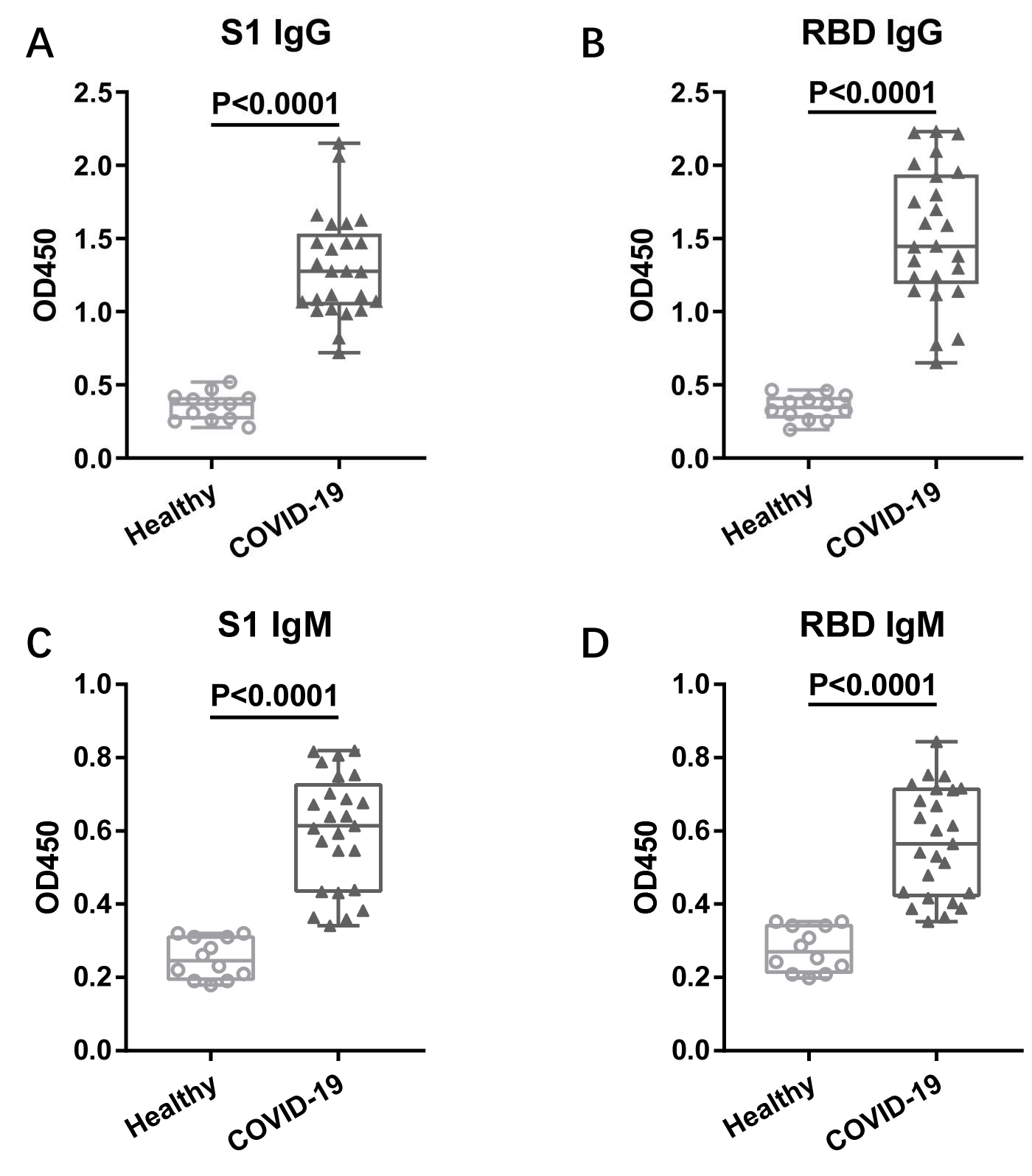

Supplement: Supplementary Figure 1 — COVID-19 Patients Developed SARS-CoV-2 Specific Antibodies. Comparison of The IgG and IgM antibody titer against S1 protein or RBD between COVID-19 patients (n=25) and healthy people (n=13). The antibody titers of COVID-19 patients were collected two weeks after symptom onset. [file Image_1.tif]

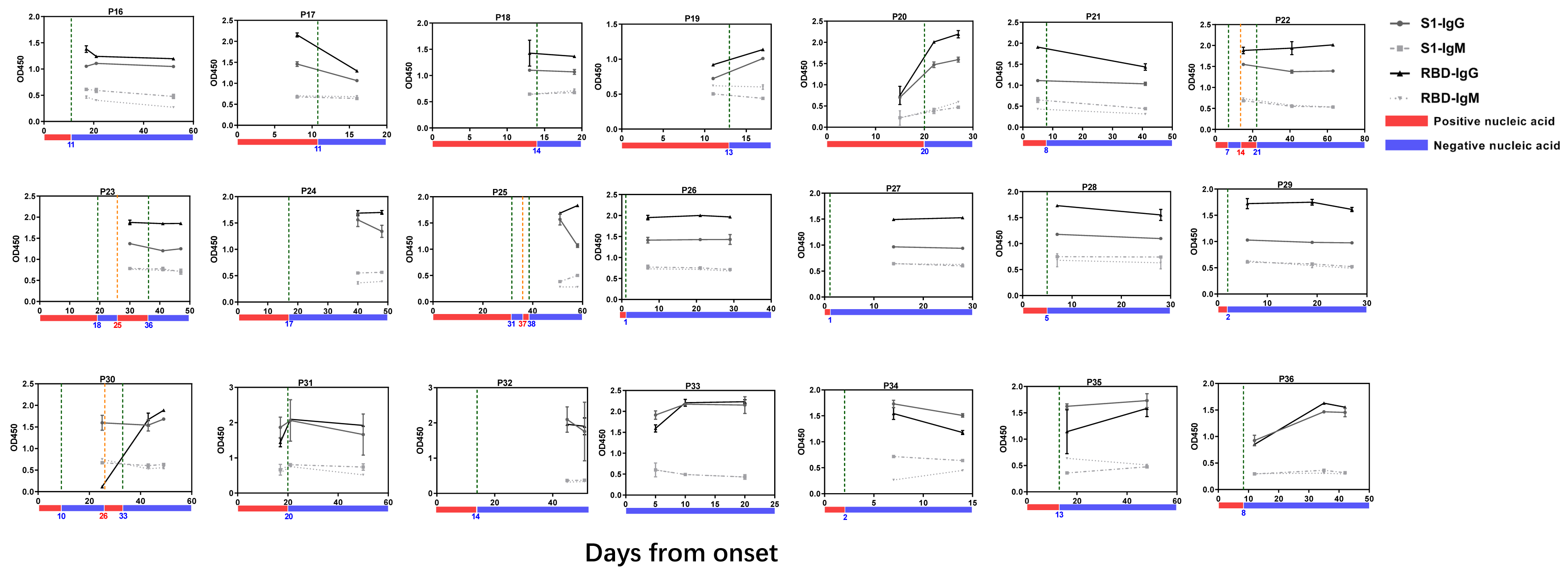

Supplement: Supplementary Figure 2 — Detection of IgG and IgM Antibody against S Protein or RBD in COVID-19 Patient. Plasma were collected at different time points after syndrome onset. The titers of IgG or IgM antibody binding to S1 protein or RBD in 21 COVID-19 patient plasma are shown. The green dotted line represents the time point when the patient’s nucleic acid turns negative while the orange dotted line represents the time point when the patient’s nucleic acid turns positive. The red process bar indicates that the patient’s nucleic acid is positive, and the blue indicate a negative result. [file Image_2.tif]

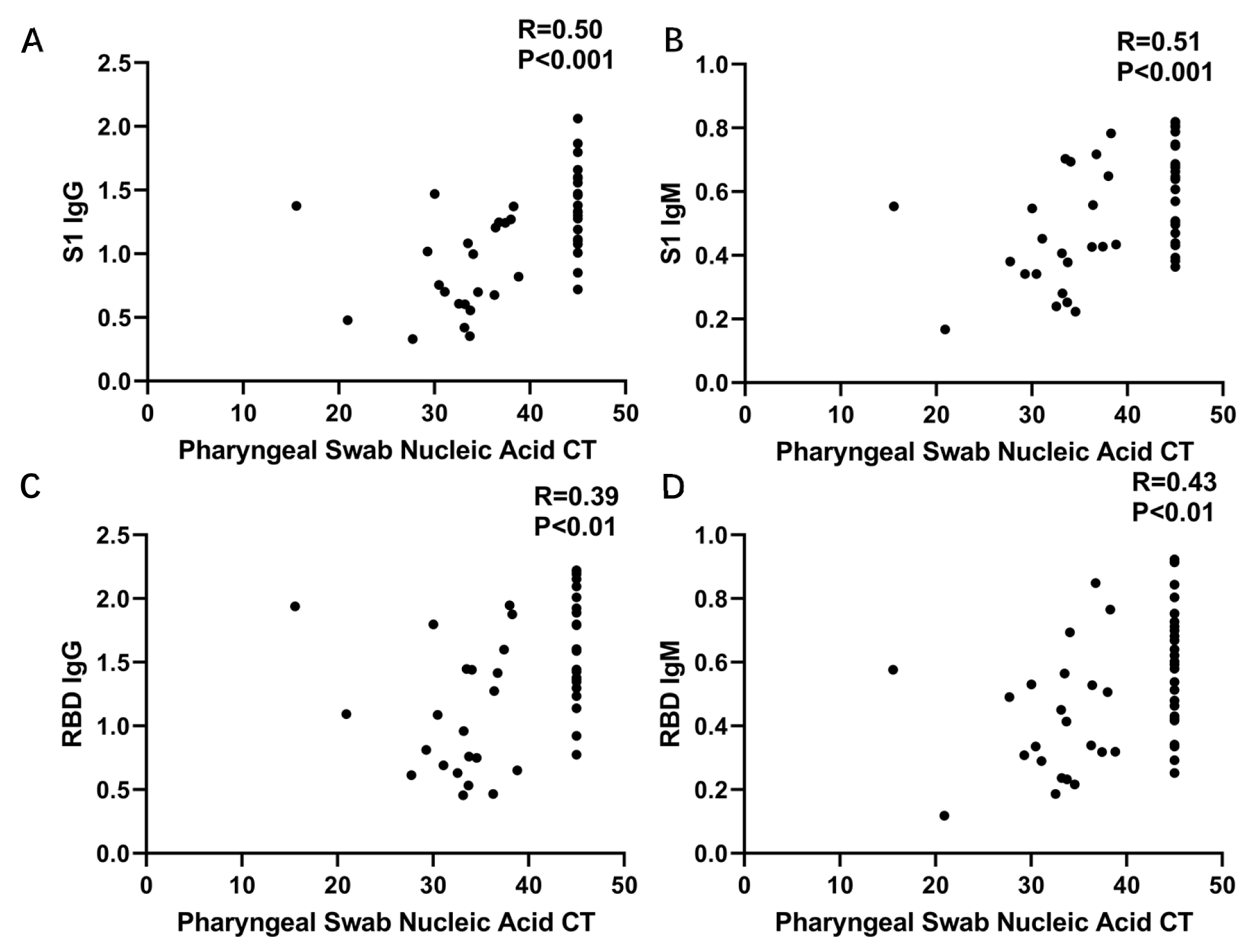

Supplement: Supplementary Figure 3 — The Nucleic Acid Ct Value in Throat Swab was Positively Correlated with the Anti-S and Anti-RBD IgG and IgM Antibody Titer. The titers of IgG or IgM antibody against S1 protein or RBD between 8-21 days from symptom onset and the nucleic acid Ct value in throat swab of each patient during their hospitalization period were collected. Fifty samples were included in this figure. [file Image_3.tif]

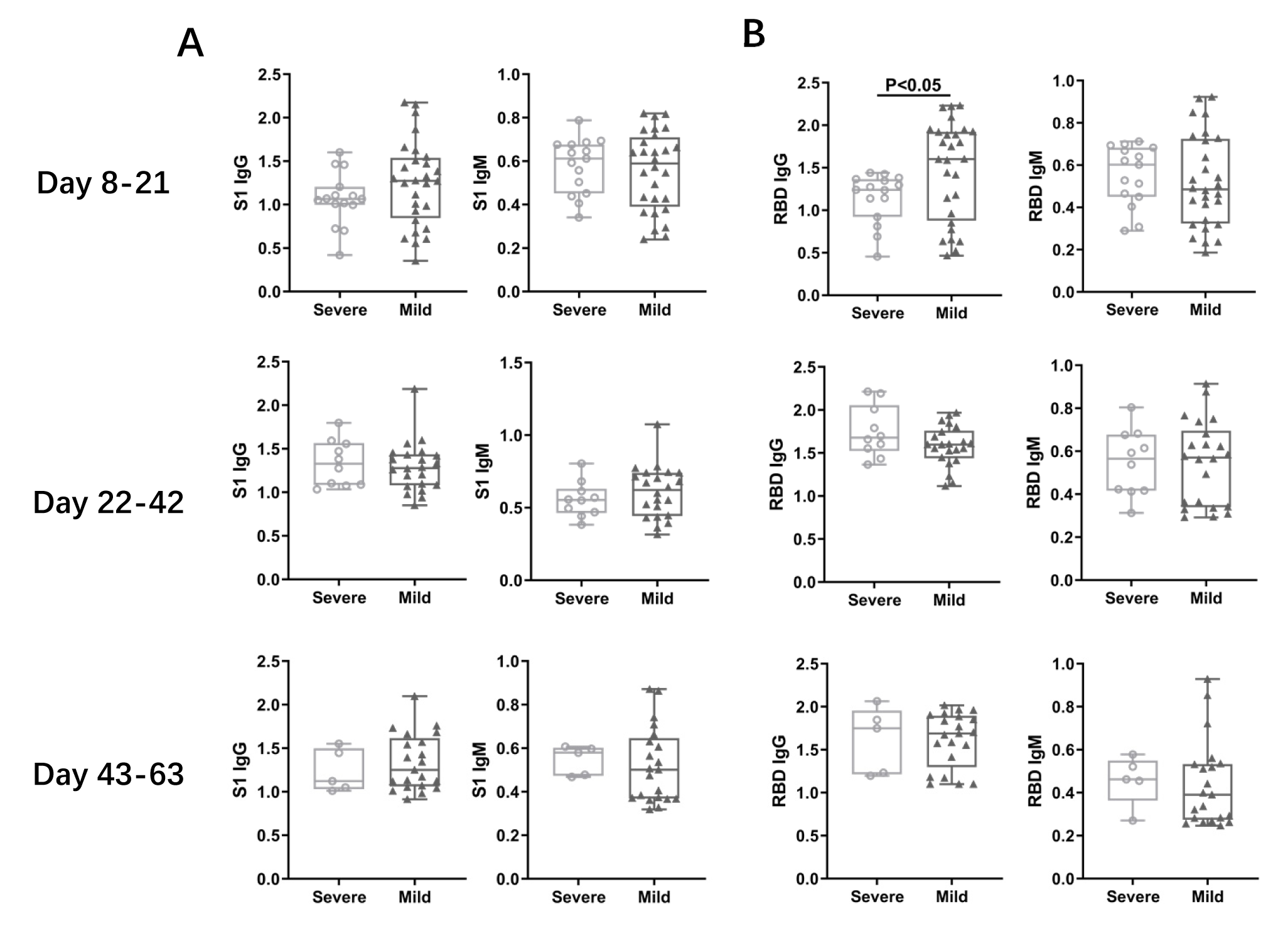

Supplement: Supplementary Figure 4 — The anti-S1 or anti-RBD IgG and IgM antibody titers were comparable in the severe and mild group between 8-63 days from symptom onset. The patients were grouped by disease symptom. The titers of IgG and IgM antibody against S1 (A) protein or RBD (B) between 8-63 days from symptom onset were collected and analyzed. [file Image_4.tif]
